# Mesoporous Silica-Based Membranes in Transdermal Drug Delivery: The Role of Drug Loss in the Skin

**DOI:** 10.3390/pharmaceutics16080995

**Published:** 2024-07-26

**Authors:** Frank Baumann, Theresa Paul, Susann Ossmann, Dirk Enke, Achim Aigner

**Affiliations:** 1 Rudolf-Boehm-Institute for Pharmacology and Toxicology, Clinical Pharmacology, Faculty of Medicine, Leipzig University, 04107 Leipzig, Germany; baumann-leipzig@gmx.de; 2 Institute of Chemical Technology, Leipzig University, 04103 Leipzig, Germany; theresa.paul@uni-leipzig.de; 3 Leipzig Heart Center, University Department for Cardiac Surgery, 04289 Leipzig, Germany; susann.ossmann@helios-gesundheit.de

**Keywords:** drug release, mesoporous membrane, porcine skin, drug metabolization, Franz cell, LC-MS

## Abstract

Compared to other forms of drug administration, the use of Transdermal Drug Delivery Systems (TDDSs) offers significant advantages, including uniform drug release profiles that contribute to lower side effects and higher tolerability, avoidance of direct exposure to the gastrointestinal tract, better patient compliance due to their non-invasive means of application and others. Mesoporous silica membranes are of particular interest in this regard, due to their chemical stability and their tunable porous system, with adjustable pore sizes, pore volumes and surface chemistries. While this allows for fine-tuning and, thus, the development of optimized TDDSs with high loading capacities and the desired release profile of a given drug, its systemic availability also relies on skin penetration. In this paper, using a TDDS based on mesoporous silica membranes in Franz cell experiments on porcine skin, we demonstrate surprisingly substantial drug loss during skin penetration. Drug passage through porcine skin was found to be dependent on the age and pre-treatment of the skin. pH and temperature were major determinants of drug recovery rates as well, indicating drug loss in the skin by enzymatic metabolization. Regarding the TDDS, higher loading obtained by SO_3_H surface modification of the mesoporous silica membranes reduced drug loss. Still, high loss rates in the skin were determined for different drugs, including anastrozole, xylazine and imiquimod. We conclude that, beyond the fine-tuned drug release profiles from the mesoporous silica membrane TDDS, remarkably high drug loss in the skin is a major issue for achieving desired skin penetration and, thus, the systemic availability of drugs. This also poses critical requirements for defining an optimal TDDS based on mesoporous silica membranes.

## 1. Introduction

Mesoporous silica materials have gained enormous scientific interest for their sustained drug release capabilities, and they offer several advantages over other systems, including their tunable porous system with adjustable pore sizes, pore volumes and surface chemistries, as well as their chemical stability and high biocompatibility [1,2,3,4]. Previously, we described monolithic SiO_2_-based carriers as sustained drug release systems [5]. The systematic variation in mesoporous glass properties and the subsequent analysis of structure–property relationships revealed drug loading concentrations and pore volumes, but not pore surface area or pore diameter, as the most important parameters for drug loading and the kinetics of drug release [5]. We further introduced chemically modified, low- and high-porosity mesoporous silica membranes, prepared by post-synthetic methods. Different effects of SO_3_H- (strongly acidic), COOH- (weakly acidic), CN- or OMe- surface modifications revealed the possibility of further fine-tuning and improving overall loading efficacies and release profiles, dependent on the chemical properties of a given drug [6]. While these studies for establishing structure–property relationships were performed under submerse release conditions, their potential use as Transdermal Drug Delivery Systems (TDDSs), which play important roles and offer several advantages in drug therapy (see, e.g., [7,8,9] for a review), leads to a quite substantially different situation. Beyond another microenvironment for drug release, this also applies to drug transition through the skin, including drug metabolization.

The physiological function of the skin comprises two barrier functions: a physical and a biochemical barrier [10]. As the outermost part of the skin, the stratum corneum represents the first barrier of the body against the external environment. It has been well established that foreign substances, including drugs, need to meet certain physicochemical properties for overcoming this barrier, and various strategies for enhancing drug penetration have been described [8,9,11,12,13,14,15]. The second, biological, barrier comprises enzymes, which catalyze the biotransformation of foreign substances [10]. While comparably little is known about this skin metabolism, these effects may well hamper or even preclude the dermal delivery of foreign substances, even if they are not trapped by the first barrier, and thus must not be underestimated. At first glance, metabolism largely resembles the enzymatic biotransformation in the liver, with phase I functionalization reactions prior to phase II conjugation reactions for increasing hydrophilicity and/or inactivation [16,17]. However, studies have shown that both the enzyme types and their activities differ from the liver, and, thus, skin metabolism has to be studied separately [10]. The main site of metabolization is the viable epidermis, while the dermis also shows some enzymatic activity, albeit to a lesser extent. Also, considering the shorter residence time of drugs in the dermis prior to systemic availability, the epidermis plays a major role.

Enzymes identified in the skin include different cytochrome P450 isoenzymes, with considerable variability between gender, age and the anatomical site and somewhat conflicting data regarding the expression of single isoenzymes [10]. Conflicting results may also rely on different methods of determination. Other phase I enzymes identified in the skin include alcohol dehydrogenase (ADH), aldehyde dehydrogenase (ALDH), cyclooxygenase (COX1, COX2) and Flavin-dependent monooxygenase (FMO) for oxidation, as well as carboxylesterase for hydrolyzation and others (see [16,17] and references therein). Likewise, phase II enzymes in native human skin include N-acetyltransferases (NATs), glutathione S-transferases (GSTs), UDP-glucuronosyltransferases (UGTs) and others. In total, 26 phase I enzymes and 13 phase II enzymes have been found on the protein level, with several more showing mRNA positivity [16,17].

Still, since studies on skin metabolism mainly rely on enzymes identified in the context of the liver, the relevance of these pathways or their combined metabolic activity encountered in the skin is still underexplored. In this paper, rather than focusing on specific enzymes and their activity, we analyzed drug transition through the skin by performing Franz cell experiments on porcine skin and calculating the percentages of different drugs reaching the acceptor compartment (representing systemic availability) vs. being retained or metabolized in the skin, or retained in the TDDS. In particular, we show that drug loss in the skin due to biotransformation is surprisingly high and, especially in the context of TDDSs as sustained delivery systems, may well lead to the loss of 50–70% of the parental drug prior to achieving systemic bioavailability.

## 2. Materials and Methods

### 2.1. Materials

Solvents used in this study were acetonitrile (Baker/Fisher Scienitific, Schwerte, Germany), methanol (Promochem, Wesel, Germany) and bidistilled water (Honeywell, Charlotte, NC, USA) as LC-MS grade. The reference substances, anastrozole, xylazine (HCl) and imiquimod, were obtained from TCI (Eschborn, Germany); d12 anastrozole and d9 imiquimod were from Cayman Chemical (Ann Arbor, MI, USA), and d6 xylazine was obtained from Sigma Aldrich (Taufkirchen, Germany). For LC-MS, a SpectraSystem apparatus (Finnigan/Thermo Fisher, Waltham, MA, USA) was employed, with pump (SpectraSystem P4060), degasser (SpectraSystem SCM 1000), interface (SpectraSystem SN 4000) and a single mass spectrometer SSQ7000. The software used for data evaluation was XCalibur Version 1.3 and SSQ Tune Version 1.1.

**Figure 1:**
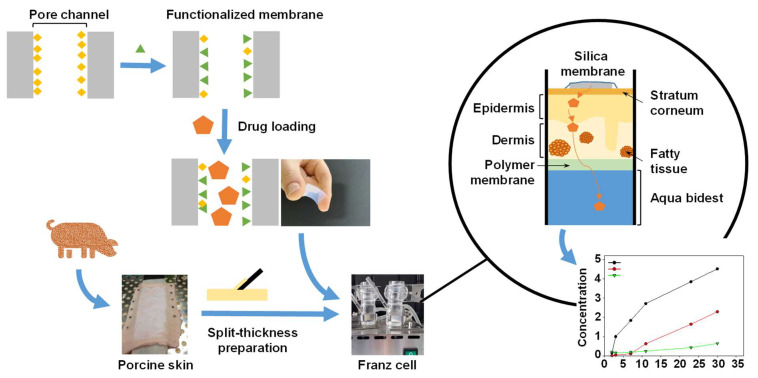
Schematic illustration of the mesoporous silica membrane and skin preparation and the Franz cell experiments.

Procedures in this study were performed according to the scheme below (Figure 1):

### 2.2. Preparation of Mesoporous Silica Membranes

The mesoporous silica membranes, including their chemical surface modification, were prepared as described previously [5]. Briefly, mixing of analytical grade silicon dioxide (SiO_2_, Alfa Aesar 99.5%), boron trioxide (B_2_O_3_, Alfa Aesar 98%), sodium carbonate (Na_2_CO_3_, Roth 99.5%) and sodium nitrate (NaNO_3_, Merck 99.99%) and melting at 1400 °C for 2 h in an electric furnace (Nabertherm LHT 04/17) yielded a 70 SiO_2_ · 23 B_2_O_3_ · 7 Na_2_O (wt%) glass. After enhancing the chemical homogeneity by crushing, grounding and re-melting the glass at 1400 °C for 2 h, it was poured on a preheated brass plate and annealed to avoid thermal stress. Glass blocks were thermally treated at 600 °C for 24 h for generating an interconnected system of a sodium-rich borate phase and a silica-rich phase and were then cut into 10 × 10 × 0.3 mm^3^ plates using a precision saw (Buehler IsoMet High Speed Pro, ITW Test & Measurement, Leinfelden-Echterdingen, Germany). For removing the sodium-rich borate phase, the plates were leached for 2 h in diluted HCl at a temperature of 90 °C. For SO_3_H functionalization, mesoporous silica membranes positioned in a Teflon basket were refluxed in 140 mL of dry toluene under nitrogen atmosphere [6]. Upon addition of 3-(Trimethoxysilyl)-1-propanethiol (3-MPTS, Alfa Aesar 95%), the suspension was refluxed at 100 °C for 20 h, prior to washing in toluene and drying. For the oxidation of the mercapto groups, the membranes were then refluxed in 140 mL of 65% HNO_3_ (Fluka 65%) for 7 h at 95 °C, prior to washing with water and drying [6].

Details on the mesoporous silica membranes can be found in the Supplementary Materials (Figure S1, Table S1 and accompanying text).

### 2.3. Membrane Loading and Drug Release

Drugs were dissolved in acetonitrile (anastrozole), methanol (xylazine) or 90% acetonitrile/1% formic acid (imiquimod) at a concentration of 10 mg/mL or 50 mg/mL. The plates were dried (100 °C for 30 min) to remove residual moisture, prior to loading the plates in a 24-well plate in a 0.5 mL solution for 6 h. After loading, membranes were gently wiped off to remove the residual solution from their surface. Prior to drug release measurements, the loaded membranes were completely dried by evaporating residual solvents at 100 °C for 15 min (acetonitrile or methanol).

### 2.4. Skin Preparation and Franz Cell Experiments

#### 2.4.1. Skin Preparation

The skin was harvested in the Leipzig Heart Center according to a standard operating procedure [18]. A distance of at least 5 cm was maintained from the spine, the ribs and the center of the abdomen on the side abdominal wall. The skin pieces were transported on ice, prior to removing adjacent muscle tissue and fat. As indicated in the Results section, skin was also obtained from a local butcher. The prepared skin pieces (approximately 10 × 10 cm^2^) were wrapped in aluminum foil and stored at −20 °C until use, for a maximum of 6 months. After thawing, the skin was stretched on a self-made device and mechanically fixed using nails. A 1 mm thick split-thickness skin was prepared using a dermatome. After carefully removing the hair, a round piece of skin (32 mm in diameter) was punched out using a hole punch, and kept in distilled water for at least 5 min. After determination of their thickness using a digital caliper, these skin pieces were then used for the experiments.

#### 2.4.2. Franz Cell Experiments

A 25 mm clear jacketed Franz diffusion cell (volume 20 mL; SES Analysensysteme, Bechenheim, Germany), equipped with a two-cell stirrer and a thermostat, was used. The skin was placed on a 32 mm polyethersulfone membrane (Dorsan, Hambrücken, Germany). The loaded TDDS (mesoporous glass membrane) was placed in the middle of the hydrated skin and fixed with a foil (3 M Sciences Applied to Life Medical Materials & Technologies, Cergy-Pontoise Cedex, France). Alternatively, where indicated, drug solutions were applied directly onto the skin. The receptor chamber of the Franz cell was filled with bidistilled water (LC-MS grade, pH 7.0) or, where applicable, 10 mM HEPES (pH 5.5) and the system was checked for the absence of air bubbles. The upper part was covered with parafilm. In total, 200 µL samples were taken from the sampling port at the time points 1, 2, 3, 7,11, 23, 30, 36, 48 and 60 h, with the addition of 200 µL of fresh volume for substitution. The samples were stored at −20 °C until measurement.

Besides the receptor chamber, drug amounts remaining in the foil, membrane and mesoporous glass membrane (TDDS) were determined as well. For this purpose, the skin, the film and the membrane were chopped, prior to adding 3 mL acetonitrile (for anastrozole or imiquimod) or methanol (xylazine) and incubating overnight. For the determination of 100% values, freshly loaded glass membranes were extracted in 3 mL of the same solvent. The extract was transferred to a glass tube, and the solvent evaporated using nitrogen. The residual mass was dissolved in the following solvents: glass membrane and membrane in 0.5 mL of water; skin in 1 mL of water and 1 mL and acetonitrile; and foil in 2 mL of water. To a 200 µL volume of each sample, 20 µL internal standard solution was added, prior to filtration in 0.22 µm centrifuge filters at 12,000 rpm.

### 2.5. Determination of Drug Concentrations and Drug Release Profiles

A single LC-MS method was used for the determination of drug concentrations. Two different solvents were used: 40% acetonitrile + 0.1% formic acid (xylazine, imiquimod) or 55% acetonitrile + 0.1% formic acid (anastrozole). For all substances, an Atlantis column (2.1 mm × 150 mm + precolumn 5 mm; Waters, Eschborn, Germany) was used, and a flow rate of 0.2 mL/min was applied. D12-anastrozole, d9-imiquimod and d6-xylazine were used as internal standards.

The drugs were measured at the following mass units (dalton): xylazine (221; internal standard: 227), anastrozole (225; 237 (fragment ions of the drug and the internal standard)) and imiquimod (241; 250). For calibration curves, see [5]. For all substances, the following parameters were used for the single mass spectroscopy: 40 psi nitrogen as sheath gas and 10 psi nitrogen as auxiliary gas, 220 °C capillary temperature, 4.5 kV ionization voltage in positive modus and 1400 V multiplier voltage. The current ionization–dissociation voltage (CID) was 10 V for xylazine and imiquimod and 20 V for anastrozole, respectively.

## 3. Results

### 3.1. Drug Skin Transition Is Dependent on the Skin and TDDS Properties

**Figure 2:**
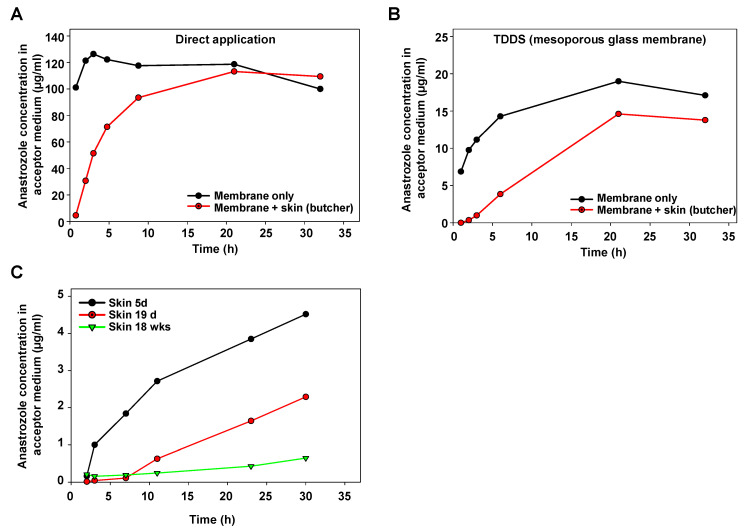
Determination of anastrozole permeation in Franz cell experiments. (**A**) Time-dependent increase in the anastrozole concentration in the acceptor medium upon direct application of the drug onto the carrier membrane (black), or with porcine skin obtained from a regional butcher (red). (**B**) Application of the drug using a mesoporous silica membrane as a TDDS, leading to a sustained release profile. Again, increase rates of anastrozole drug concentration (black) are further reduced upon drug penetration through skin (red). (**C**) Skin age-dependent barrier function for drug penetration, as determined by slower increase in anastrozole concentration in the acceptor medium over time when using skin from older pigs.

For initial characterization of the Franz cell device, anastrozole as a test drug was directly applied onto the membrane separating the upper and the lower part. A very rapid transition into the lower part (acceptor container) was observed, confirming that the membrane itself does not represent a major barrier (Figure 2A, black line). By contrast, when positioning porcine skin obtained from a butcher and prepared, as described above, onto the separating membrane, the increase in anastrozole concentration in the acceptor medium was delayed (Figure 2A, red line).

As to be expected, a substantially slower profile was obtained when switching to a mesoporous glass membrane as the TDDS (Figure 2B, black line). Again, positioning this TDDS onto porcine skin led to further retardation of the anastrozole accumulation in the acceptor fluid as compared to the membrane-only setting without skin (Figure 2B). The marked influence of the skin’s properties also became evident when directly comparing skin from pigs at different ages: while the skin from a 5 d old piglet showed some delaying effects on the transition of anastrozole released from the TDDS, this barrier function was markedly higher in the skin from a 19 d old pig, and skin penetration was further reduced in the skin from an 18-week-old pig (Figure 2C).

It should be noted that the latter experiment employed fresh skin directly prepared after pig euthanasia, rather than skin from the butcher with (heat) pretreatment. Thus, beyond barrier functions due to porcine skin anatomy, these results and, in particular, the direct comparison between freshly prepared skin vs. skin from a local butcher also indicated biochemical processes—in particular, enzymatic metabolization—as additional factors. Based on these findings, drug loss in the skin during drug penetration was further studied.

### 3.2. Drug Loss Is Dependent on External Conditions during Skin Transition

**Figure 3:**
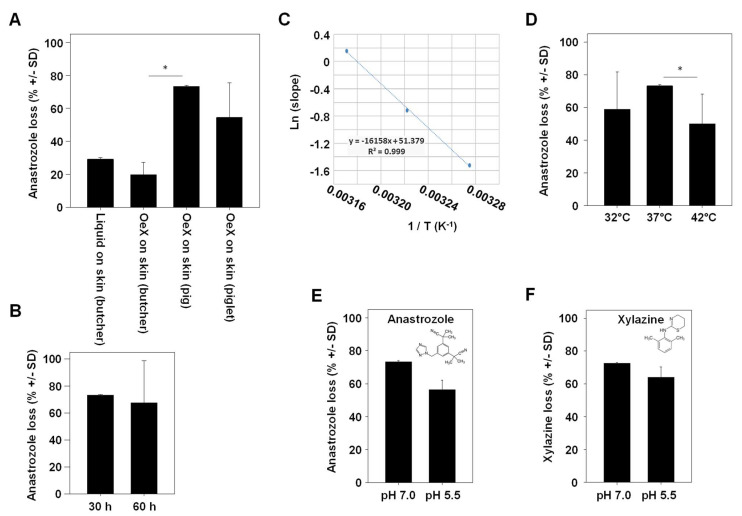
Determination of variables affecting anastrozole drug loss upon skin penetration. (**A**) Comparison between adult porcine skin obtained from a local butcher (with heat pretreatment) vs. native skin from a piglet or an older pig. OeX: a mesoporous silica membrane acting as a TDDS. (**B**) Comparison of anastrozole loss over two different experimental durations. (**C**) Arrhenius plot demonstrating the direct correlation between temperature (32 °C, 37 °C and 42 °C in the Franz cell) and the permeation rate. (**D**) Temperature-dependency of drug loss upon skin permeation, with highest loss observed at a physiological 37 °C. (**E**,**F**) pH-dependence of (**E**) anastrozole or (**F**) xylazine drug loss. * indicates statistical significance.

In the subsequent experiment, ‘drug loss’ in our Franz cell experiments was defined as the percentage of the drug that could not be found unmetabolized in any of the compartments (glass-membrane TDDS, acceptor compartment, separating membrane and porcine skin). When employing skin obtained from a butcher, a ~25–30% loss of anastrozole was observed. Values were largely independent of whether the drug was applied directly onto the skin or whether a glass-membrane TDDS without chemical modification (‘OeX’) was used (Figure 3A, left bars).

Notably, anastrozole loss was markedly higher when switching to native skin, with a ~50% loss in the case of piglet skin and an even higher ~ 70% loss in the case of skin from an older pig (18 weeks; Figure 3A, right bars). The total duration of the experiment was found to have no effect on overall drug loss (Figure 3B). By contrast, the temperature was critical in two ways, as shown by measurements at a physiological 37 °C vs. above or below physiological conditions (42 °C or 32 °C, respectively). This covers the range of major biological relevance, with 37 °C being physiological temperature, 42 °C marking the upper limit (life-threatening) temperature and 32 °C representing medium hypothermia. On the one hand, the Arrhenius plot showed a direct and linear dependence of the drug permeation rate on the temperature (Figure 3C), while on the other hand, percentages of drug loss were highest at 37 °C (70%) and decreased to ~50% at higher (42 °C) or lower (32 °C) temperatures (Figure 3D). This indicates, beyond the well-established effect of temperature on skin permeation processes, the role of enzymatic metabolization of the drug as a key mechanism in drug loss. The same was true when switching from a physiological pH of 7.0 to a lower pH of 5.5 (Figure 3E). Notably, results almost identical to anastrozole were obtained for the sedative, analgetic, anesthetic and muscle-relaxing drug xylazine, despite its different chemical structure (Figure 3F).

### 3.3. Percentage of Drug Loss during Skin Transition Is Decreased with Higher TDDS Loading

**Figure 4:**
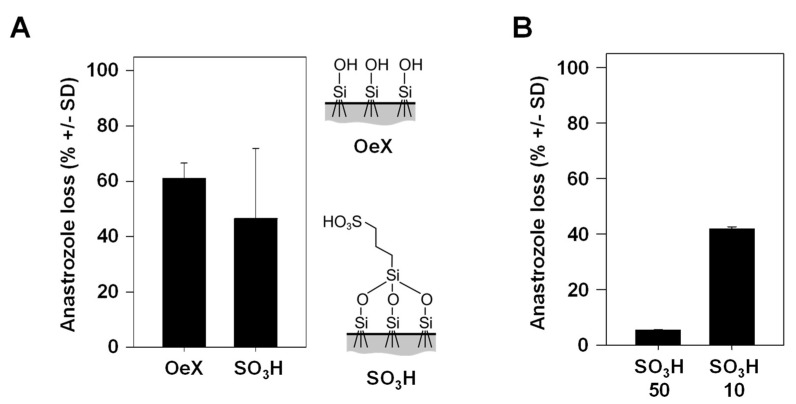
Anastrozole drug loss dependence on the properties of the mesoporous silica membrane acting as a TDDS. (**A**) Comparison of drug loss between an unmodified (OeX) and a chemically modified (SO_3_H) mesoporous silica membrane. Right: scheme of the chemical surface structures. (**B**) Markedly reduced drug loss during skin penetration upon loading of the chemically modified (SO_3_H) mesoporous silica membrane with 5-fold larger anastrozole amounts (50 mg/mL vs. 10 mg/mL, referring to the concentration of the solution in the initial loading process).

Previously, we introduced SO_3_H-modified mesoporous glass membranes as TDDSs with higher drug-loading capacity as compared to their counterparts without chemical modification [6]. More specifically, non-covalent interactions of anastrozole with the negatively charged surface on the membrane and, in particular, within the pore leads to more efficient interactions, resulting in stronger drug adsorption, higher drug loading and a further sustained drug release profile compared to unmodified glass membranes. A trend towards lower drug loss was observed when comparing the SO_3_H-modified mesoporous glass membrane, associated with higher drug dosage, with its chemically unmodified (OeX) counterpart (Figure 4A). In the context of the above observation that the incubation time in the experiment plays an overall minor role in drug loss (see Figure 3B), this suggests that the slower drug release profile of the SO_3_H-modified TDDS may not significantly contribute to the difference, while the overall amounts of the loaded (and released) drug seem to be relevant. Indeed, when comparing identical mesoporous membranes loaded under standard conditions (soaking in a 10 mg/mL anastrozole solution) to its counterpart loaded at a 5-fold higher concentration, the 50 mg/mL loaded TDDS showed markedly reduced drug loss (Figure 4B). This indicates that total drug amounts are a major determinant for the percentage of drug loss and again indicates that enzymatic metabolism is an underlying cause, showing saturation kinetics at larger drug amounts.

### 3.4. Drug Loss Occurs Comparably with Different Drugs

**Figure 5:**
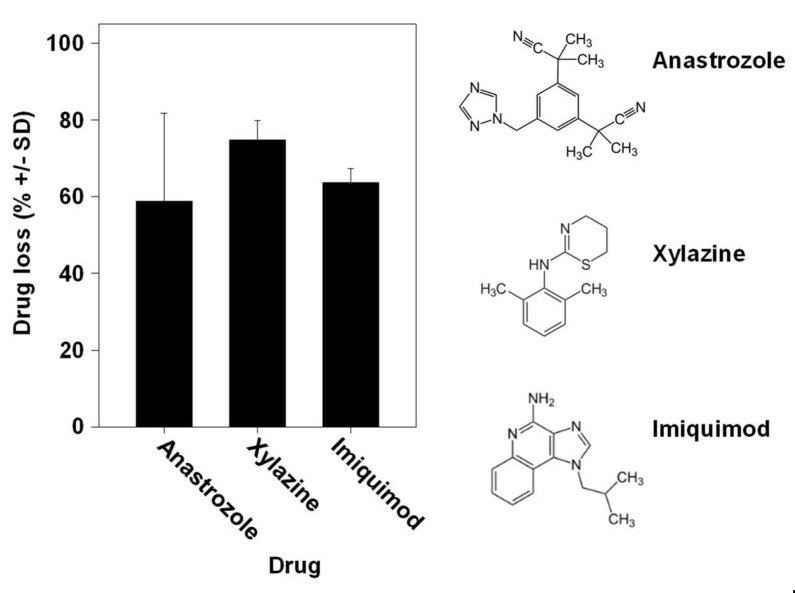
Comparison of drug loss between different drugs during skin permeation. Similar drug losses upon application of the TDDS containing the aromatase inhibitor anastrozole, the veterinary drug xylazine used for sedation, anesthesia and muscle relaxation or the chemotherapeutic imiquimod for topical application. Right: chemical structures of the drugs employed in the experiments.

Especially when considering a given defined enzymatic reaction for drug metabolization, one could expect major differences between different drugs. Interestingly, however, we found the percentages of drug loss to be very comparable between the aromatase inhibitor anastrozole, the veterinary drug xylazine used for sedation, anesthesia and muscle relaxation and the chemotherapeutic drug imiquimod for topical application, despite their differences in chemical structure and putative or known pathways of metabolization (Figure 5). This clearly indicates the parallel activity of many enzymes.

## 4. Discussion

The dermal administration of drugs is confronted with the skin as an efficient barrier against foreign compounds. While strategies for the improvement of drug penetration have largely focused on the stratum corneum and overcoming this first mechanical skin barrier so far, the second (biological) skin barrier is largely underestimated, despite the fact that the first evidence of compound metabolism was already described more than seventy years ago (see [16]). While expression studies have demonstrated the presence of metabolizing enzymes on the mRNA and/or protein level, metabolic activities in ex vivo skin are often found to be very low compared to the liver [10]. Even when requiring sensitive analytical techniques for metabolite detection in an experimental setting, this does not mean that these processes are irrelevant. This notion is supported by our findings, with, in some cases, up to 70% drug loss upon skin transition. For our experiments, we used the Franz diffusion cell [19] as a well-established static cell model. Notably, possible limitations with regard to the maintenance of skin viability beyond 24–48 h, mechanical impairment of the skin in the experimental process or microbial contamination did not seem to interfere with our measurements.

In contrast to skin homogenates or 3D skin reconstructs, the porcine skin preparations employed here contain all cell types, including keratinocytes, melanocytes, fibroblasts, endothelial and Langerhans cells, as well as hair follicles and sweat glands, and they largely retained the architecture of intact skin. Since porcine skin shows characteristics similar to those of human skin and since the genetic similarity between pigs and humans is estimated to be around 85–90%, porcine material is expected to quite closely resemble the enzymatic properties of human skin, without limitations with regard to availability. Notably, the preparation process of the split-thickness skin employed here avoided any physical (heat), chemical or enzymatic treatment, thus preserving enzymatic activity. The direct comparison of freshly obtained skin with (heat-) pretreated skin from a local butcher in our study emphasizes that avoidance of higher temperatures is critical; the previously described large abolishment of enzymatic activity upon freeze–thawing [20] was not observed in our experiments.

Alternatively, different 3D skin reconstructs have been introduced (see [21,22,23] for review). While skin constructs resemble normal healthy skin with regard to histological morphology, differences are seen on the molecular level, and external factors like the composition of the culture medium and its supplements may well affect enzymatic activities. This does not apply to intact skin preparations as used here; however, it still remains to be seen to what extent reconstructed skin may yield similar qualitative and quantitative results on drug metabolization. Beyond phase I and phase II enzymes, these studies may also have to involve transporters in the skin, as well as the assessment of polymorphisms and other possible interindividual genetic variabilities. While those are well-established parameters in drug pharmacokinetics upon systemic application, they are largely understudied in the skin. Again, our results demonstrate that experimental settings, as used here, should be able to provide important insights into these metabolization processes, which pose a major and so far under-estimated limitation for dermal drug delivery and systemic bioavailability.

Nevertheless, the limitations of the study should be considered when interpreting the findings. These include the use of porcine skin as a model for human skin (despite the large similarities; see above) and the still-limited drug selection. Future work will also have to cover in vivo studies and the assessment of transporters and genetic variabilities. A comparison with other drug delivery systems and long-term stability assessments will be of relevance as well. Thus, further research is needed to address these gaps in knowledge.

Still, based on our findings, four points may be worth considering in attempts to reduce drug loss. (i) In some cases, it may be a feasible strategy to combine the drug loaded in the TDDS with inhibitors of enzymatic metabolism, e.g., CYP inhibitors. While the systemic levels of such inhibitors may be sufficiently low upon transdermal application so as to not exert systemic side effects, this strategy may well prevent local metabolization of the drug at its site of application, i.e., in the skin. When pursuing this strategy, mesoporous silica membranes may prove particularly advantageous for two reasons: (a) their chemical inertness (i.e., allowing for the loading of compounds with a broad range of chemical properties without the risk of unwanted interaction with the TDDS) and (b) their high loading capacities. Previous studies have identified different enzymes and enzyme families that are known, e.g., from liver metabolism, to be present in the skin as well. Studies on their abundance, expression levels and contribution to enzymatic metabolism in the skin, however, are rather rare, with sometimes conflicting results (see, e.g., [10,16,17]). Thus, from a skin perspective, it may be somewhat difficult to identify rate-limiting enzymes in the context of the systemic availability of a given drug after its transdermal application. The metabolization of a drug during skin penetration may not rely on a single enzyme but rather on a complex pattern of different enzymatic activities in the skin that may be more or less relevant in the case of a given drug. This will, thus, not allow for a one-size-fits-all solution when it comes to enzyme inhibition; rather, appropriate inhibitors will have to be selected for each individual drug. However, considering the chemical structure of a given drug, it may well be possible to short-list enzymes relevant for its metabolization. Despite the enzymatic differences between the liver and skin, efforts can benefit from previous knowledge on the hepatic metabolization of a drug of interest.

Age may play a role as well, and beyond the, perhaps not surprising, age dependence of the physical skin barrier, we also found age-dependent enzyme-related drug loss. It is well known in pharmacology that in the case of several drugs, dosages need to be adjusted in children and/or in older patients, considering major differences in enzymatic drug transport and metabolization capacities. It is reasonable to conclude that this also applies to transdermal application, suggesting the need of TDM (therapeutic drug monitoring) studies after the transdermal application of a given drug to address this aspect. From an ethical viewpoint, however, this may encounter limited feasibility in certain age groups (e.g., children). Again, this highlights the relevance of preclinical ex vivo studies, as performed here, to further elucidate to what extent a given drug may be affected. This may well help rationally identify appropriate drug dosages in a given patient (or group), also in the context of very young or old age.

(ii) Alternatively, it is possible to consider just applying larger drug amounts in the TDDS, compensating for the anticipated drug loss during skin penetration. Again, this strategy may benefit from the comparably high loading capacities of our mesoporous silica membranes as TDDSs, esp. upon introducing chemical modifications like SO_3_H. (iii) Certain drugs may be applied as prodrugs, relying on their activation in a biological environment. In pharmacology, this strategy is already well established for several enteral or parenteral drugs. Based on our findings, however, it may be further explored in drugs for transdermal application. While this concept has been described previously (see [10] and references therein), our data highlight that the relevance and efficacy of this approach may possibly be higher than previously thought, considering the high levels of metabolization. (iv) Finally, in the context of drug development, where physical drug properties favoring skin penetration must be kept in mind when transdermal application is desired, drug stability against the rather extensive metabolization in the skin may become an even more central molecular property, and it should be kept in mind that metabolizing enzymes and metabolic pathways may differ from the liver, as described previously and discussed above.

## 5. Conclusions

Our findings highlight that, beyond the release profiles from a given TDDS and the barrier function of the skin, drug loss in terms of drug metabolization plays a major role in determining the systemic availability of a given drug. Considering that, under many circumstances, > 50% drug loss was observed, this is a major limitation of TDDS-based drug delivery. While permeation enhancers are already in use for addressing the physical barrier function of the skin, the parallel application of inhibitors, e.g., of phase I and/or phase II enzymes, should be considered as well, since metabolization in the skin was found to be surprisingly high. From a TDDS perspective, the use of mesoporous glass systems may be of particular advantage since these are chemically widely inert and thus compatible with many compounds to be (co-)delivered. From a drug perspective, the skin must be considered an organ with perhaps surprising metabolic activity, thus requiring concepts for (local) enzyme inhibition. This work highlights the need for this strategy and may provide the basis for novel TDDS systems.

## Data Availability

Data will be made available on request.

## Supplementary Materials

The following supporting information can be downloaded at https://www.mdpi.com/article/10.3390/pharmaceutics16080995/s1, Figure S1: Scheme of the pore surface, functionalized with 3-(Trimethoxysilyl)-1-propanethiol (MPTMS; left), pore width distribution of a membrane functionalized with MPTMS (center) and scanning electron microscopy (SEM) picture of a porous silica membrane, with arrows indicating colloidal silica species that form the 9 nm pores inside the pore system of porous silica with 50 nm pore size [24] (right); Table S1: Textural properties of unmodified and chemically functionalized silica membranes, analyzed by N_2_-sorption and CHN-elemental analysis.
