# Supplementary material for: Mesoporous Silica-Based Membranes in Transdermal Drug Delivery: The Role of Drug Loss in the Skin
Source: Pharmaceutics. 2024 Jul 26;16(8):995. doi: 10.3390/pharmaceutics16080995 (PMC11358937; doi:10.3390/pharmaceutics16080995)
Supplement: Supplementary file 1 [file pharmaceutics-16-00995-s001.zip › pharmaceutics-3093422-supplementary.pdf]

Supplementary Materials

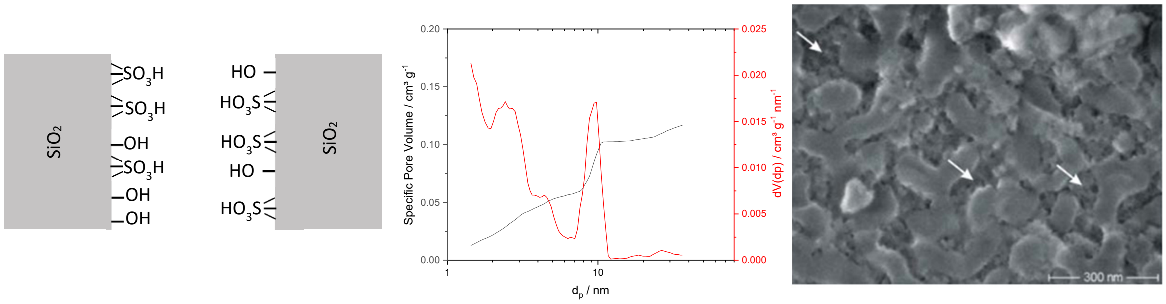

Figure S1: Scheme of the pore surface, functionalized with 3-(Trimethoxysilyl)-1-propanethiol (MPTMS; left), pore width distribution of a membrane functionalized with MPTMS (center) and scanning electron microscopy (SEM) picture of a porous silica membrane, with arrows indicating colloidal silica species that form the 9 nm pores inside the pore system of porous silica with 50 nm pore size [1] (right).

Table S1: Textural properties of unmodified and chemically functionalized silica membranes, analyzed by N_2-sorption and CHN-elemental analysis.

	BET surface area (m^2/g)	Pore volume (cm^3/g)	Pore width (nm)	Surface coverage (molecule*nm^{-2})
OeX	137	0.15	9	
SO_3H	122	0.11	9	2.2

Porous silica membranes are > 98 % silica materials, that show an interconnected pore structure in the mesopore range. A secondary phase of colloidal silica is found in the primary pore system (see above, Figure S1 right), resulting in a broad pore width distribution of < 9 nm and a masked pore volume of 0.15 cm^3/g [1]. The subsequent functionalization with MPTMS shows no effect on pore size, but pore volume and specific surface area are slightly reduced. The organic chains of MPTMS have a smoothing effect on the surface area and constrict the pore system further. In addition to the -SO_3H species, unreacted silanol groups still exist on the pore surface (Figure S1, left). The textural properties were analyzed by nitrogen physisorption, whereby the samples were degassed at 250 °C for 10 h (or 80 °C for 16 h in case of functionalized membranes). The analysis was performed on Quantachrome autosorb iQ (Quantachrome Instruments, Anton Paar QuantaTec, Boynton Beach, FL, USA) at a temperature of -196 °C in the relative pressure range of 0 - 0.995 p/p_0. The pore width distribution was assessed by NLDFT modelling for silica materials with cylindrical pore shape.

Surface coverage was analyzed by CHN-elemental analysis, using Vario EL III (Elementar Analysensysteme, Langenselbold, Germany), whereby the concentration of functional groups on the surface was calculated by carbon content c(C) in relation to the specific surface area A_{BET}.

$$\frac{N_x}{nm^2} = \frac{c(C) \cdot N_A \cdot 10^{-18}}{M_C \cdot 100\% \cdot A_{BET} \cdot x_C}$$

N_A ….. Avogadro constant X_c …… number of carbon atoms in MPTMS
M_C …. molar mass of carbon

[1] D. Enke, K. Otto, F. Janowski, W. Heyer, W. Schwieger, W. Gille, Journal of Materials Science, 2001, 36, 2349.
